# Efficient NFS Model for Risk Estimation in a Risk-Based Access Control Model

**DOI:** 10.3390/s22052005

**Published:** 2022-03-04

**Authors:** Hany F. Atlam, Muhammad Ajmal Azad, Nawfal F. Fadhel

**Affiliations:** 1 School of Computing and Engineering, University of Derby, Derby DE22 1GB, UK; m.azad@derby.ac.uk; 2 Computer Science Engineering Department, Faculty of Electronic Engineering, Menoufia University, Menouf 32952, Egypt; 3 Electronic and Computer Science Department, University of Southampton, Southampton SO17 1BJ, UK; nawfal@soton.ac.uk

**Keywords:** risk estimation, NFS model, Internet of Things, security risk, risk-based access control

## Abstract

Providing a dynamic access control model that uses real-time features to make access decisions for IoT applications is one of the research gaps that many researchers are trying to tackle. This is because existing access control models are built using static and predefined policies that always give the same result in different situations and cannot adapt to changing and unpredicted situations. One of the dynamic models that utilize real-time and contextual features to make access decisions is the risk-based access control model. This model performs a risk analysis on each access request to permit or deny access dynamically based on the estimated risk value. However, the major issue associated with building this model is providing a dynamic, reliable, and accurate risk estimation technique, especially when there is no available dataset to describe risk likelihood and impact. Therefore, this paper proposes a Neuro-Fuzzy System (NFS) model to estimate the security risk value associated with each access request. The proposed NFS model was trained using three learning algorithms: Levenberg–Marquardt (LM), Conjugate Gradient with Fletcher–Reeves (CGF), and Scaled Conjugate Gradient (SCG). The results demonstrated that the LM algorithm is the optimal learning algorithm to implement the NFS model for risk estimation. The results also demonstrated that the proposed NFS model provides a short and efficient processing time, which can provide timeliness risk estimation technique for various IoT applications. The proposed NFS model was evaluated against access control scenarios of a children’s hospital, and the results demonstrated that the proposed model can be applied to provide dynamic and contextual-aware access decisions based on real-time features.

## 1. Introduction

Traditional access control models, while successful in solving various problems in some situations, are designed to offer a link between information associated with an access control rule logic and a resource to which access is requested. The implementation of an access control model can be manipulated in a variety of ways, ranging from an unforeseen situation involving poorly designed access policies to malicious entities gaining access to a group of existing accounts. As a result, traditional access control models, which are based on static and predefined policies, cannot manage unanticipated scenarios and situations [1]. As a result, they are incompatible with a dynamic and distributed system like the IoT. Instead, the IoT system requires a dynamic access control model. Dynamic access control models are based on the idea that they use not only access policies, but also dynamic and contextual features that are estimated at the time of the access request to make access decisions [2]. This provides more flexibility and can adapt to varying situations and conditions while making access decisions.

Dynamic access control techniques, as opposed to static policies, make access decisions based on real-time and contextual features. Trust, risk, context, history, and operational needs are examples of real-time features [3,4]. A risk-based access control model is one of the dynamic models that perform a risk analysis on each access request to permit or deny access requests dynamically [5,6].

The risk estimation process is an important stage for implementing a risk-based access control model. This process is based on calculating the likelihood of data leakage and the value of that data. The primary goal of the risk estimation process is to devise a method for ranking risks in order of priority and using risk numeric values to make access decisions in a given situation [7,8,9]. Several researchers used various risk assessment and management strategies; however, the majority of these methods relied on qualitative indicators. Risk estimation without enough data to define its likelihood and impact is like predicting the future; therefore, quantifying security risk, particularly in the context of access control, is extremely difficult.

The fuzzy logic system was one of the risk estimation techniques offered in the literature to overcome the lack of datasets for estimating the security risk value associated with access requests. As a result, the authors put the fuzzy logic system into action, and the findings showed that it generates correct and realistic risk values for access control operation, as presented in [9]. The fuzzy logic system, on the other hand, has several drawbacks. The scalability of the fuzzy logic system seems to be questionable because estimating the security risks of access control activities takes a long time. The fuzzy logic system also cannot learn or adapt to new environments, which will be a huge challenge for a dynamic and distributed system like the IoT. Therefore, this paper proposes the Neuro-Fuzzy System (NFS) model to resolve issues associated with the fuzzy logic system.

This paper proposes a novel NFS model to build the risk estimation process in the risk-based access control model for the IoT system. The proposed NFS model has been implemented to evaluate security risk values associated with each access request. The results demonstrated that the NFS model provides an efficient and accurate risk estimation technique that can adapt to the changing conditions in the IoT environment. The proposed NFS model was evaluated against access control scenarios of a children’s hospital. Using the contextual and real-time features involving time and location, the results demonstrated that the proposed NFS model can be applied to provide dynamic and contextual-aware access decisions.

The contribution of this paper can be summarized as follows:

- Proposing the NFS model to overcome flexibility and scalability issues associated with the fuzzy logic system for the risk estimation in the risk-based access control model;
- Identifying and implementing the most effective learning algorithm for the NFS model by comparing three different learning algorithms;
- Evaluating the accuracy and applicability of the proposed NFS model through providing access control scenarios of a children’s hospital.

The remainder of this paper is organized as follows: Section 2 presents related work, Section 3 provides an overview of the NFS technique, Section 4 presents the risk-based access control model, Section 5 presents the implementation of the proposed NFS model, Section 6 presents experimental results, Section 7 presents the evaluation of results, and Section 8 is the conclusion.

## 2. Related Work

Risk-based access control models are used primarily to provide the required flexibility to the access control process. Several researchers discussed creating a risk-based access control model to overcome flexibility and the inability to handle unpredicted situations. McGraw [10] introduced the Risk-Adaptable Access Control (RAdAC) model, which is based on assessing security risks and operational needs for granting or denying access. This model is used to calculate the risk associated with an access request and then compare that risk to the access control policy. The system then verifies the operational needs. If the corresponding operational needs and policy are met, access is granted. However, this model does not provide details about how to estimate risk and operational needs quantitatively. Furthermore, Khambhammettu et al. [11] developed a risk-based model based on object sensitivity, subject trustworthiness, and the difference between them. However, the model does not include how to estimate the risk quantitatively. Furthermore, this model requires a system administrator with broad experience to provide a reasonable value for each input in the early stage of the risk assessment process.

Chen et al. [12] presented a dynamic risk-based access control model for Cloud Computing. It combines the attribute-based access control model with the risk–trust assessment method. The model drives a threshold risk value from historical records to determine the access decision. However, this model lacked contextual features and the ability to learn and adapt to unpredicted situations. Furthermore, Choi et al. [13] presented a framework for a context-sensitive risk-based model for medical information systems. This framework categorizes information to calculate the risk value and apply the risk through treatment-based permission profiling and specifications. This framework provides the access decision based on the severity of the context and treatment. However, this model does not include how to estimate the risk quantitatively. Furthermore, the model is limited to medical information systems.

The essential step to developing a risk-based access control model is the risk estimation process, which assesses the risk value associated with each access request. This calculated risk value is then utilized to decide on whether to grant or deny access. It is difficult to assign a quantitative or numeric value to risk without a dataset to characterise its likelihood and impact. The fuzzy logic system was one of the risk estimation strategies provided by the literature to overcome the lack of datasets. For instance, Chen et al. [5] designed a fuzzy multi-level security model using the fuzzy logic system. The difference between the object and subject security levels is used to calculate risk. As a result, the risk value will be high if the difference is large. The output risk is expressed as a binary value of 0 (allow) or 1 (deny). The authors, on the other hand, did not explain how to calculate risk quantitatively or how fuzzy rules were created. The authors also failed to discuss the fuzzy logic system’s scalability, incapacity to learn, and time overhead concerns.

Ni et al. [14] used a fuzzy logic system to assess security risks. The risk value is calculated using subject and object security levels. However, the suggested approach faces numerous scaling issues, as estimating the security risk value takes a long time, especially with an increasing number of input parameters and fuzzy rules. The authors also did not discuss how fuzzy rules were created. In addition, Li et al. [15] developed a fuzzy modelling technique for determining the security risks of a healthcare information system. Using action severity, risk history, and data sensitivity, this model calculates the risk associated with each access request. The authors, on the other hand, did not provide any information on how to objectively evaluate risk values. Besides, it necessitates prior knowledge of diverse environmental scenarios to construct fuzzy rules. Furthermore, Diep et al. [16] presented a dynamic and flexible risk-based model by collecting useful information from the environment to make access decisions. The risk value was evaluated using outcomes of actions in terms of availability, confidentiality, and integrity. However, the model does not cover how values of outcomes of actions can be evaluated quantitatively.

Based on the recommendations of the literature review, the fuzzy logic system was implemented in [9] to resolve the unavailability of datasets and provide a quantitative and a numeric risk value for each access request to determine the access decision. Although the fuzzy logic system provides accurate and realistic risk values to implement the risk-based access control model, the major issue associated with it was the scalability and inability to learn. An access control model for the IoT system is intended to serve hundreds or thousands of users. Based on the experimental results discussed in [9], the risk estimation using the fuzzy logic system requires 57.38 s to estimate the security risks of 1000 access requests. This response time is efficient for a small network of devices, but with the IoT system, there are thousands of devices per network. This number of IoT devices is constantly increasing, which requires taking the scalability of the risk estimation technique into account and providing the required solution to resolve this issue. In addition, the risk estimation technique cannot learn or adapt to a new environment.

Therefore, the solution was to integrate an Artificial Neural Network (ANN) with the fuzzy logic system which creates NFS. NFS is an ANN technique, which is functionally equivalent to the fuzzy logic system. It combines the parallel computation and learning capabilities of an ANN with the human-like knowledge representation and explanation abilities of the fuzzy logic system. As a result, the ANN becomes more transparent, while the fuzzy logic system becomes capable of learning. In addition, the NFS can be trained to develop IF-THEN fuzzy rules and determine membership functions (MFs) for input and output variables of the system [17,18].

Among all methods of integrating fuzzy systems and ANNs, a hybrid NFS has the most potential. This is because it makes the best use of the advantages of fuzzy systems and ANNs. Hybrid systems have the property of constantly being treated as systems of fuzzy rules with ANNs used to tune MFs in preconditions and conclusions of rules based on the set of learning. A NFS has been utilized to estimate risk in various domains. For example, Kristjanpoller and Michell [19] utilized it for combining external factors to estimate the risk of a stock market in the Latin American region and improve the forecast accuracy. Conversely, Beinarovica et al. [20] used the NFS model in transportation control to improve safety by evaluating the risk of accidents using intelligent infrastructure devices. The model was used to simulate various scenarios, analyse risks, and recommend possible infrastructure or vehicle changes to reduce the probability of future disasters. Furthermore, Shahzadi et al. [21] adopted the NFS model to reduce security risks in cloud computing by identifying various risks associated with cloud computing. Furthermore, Kaur et al. [22] utilized the NFS model to improve authentication in mobile devices. The authors used the NFS model to build an implicit authentication system based on behavioural data collected for 12 weeks from different android users. Table 1 shows the summary of related work.

One of the major issues to develop an efficient and effective risk-based access control model is to create an accurate and reliable risk estimation technique that can resolve issues associated with existing techniques in terms of scalability and inability to learn. This paper utilizes the NFS model to add the learning capability to the risk estimation technique to build a dynamic and efficient risk-based access control model for IoT applications. To the best of the authors’ knowledge, no research exists that utilizes the NFS model in risk-based access control models. Therefore, this paper provides a novel NFS model to implement the risk estimation technique of the risk-based access control model.

## 3. An Overview of NFS

NFS is the result of integrating an ANN with the fuzzy logic system. It integrates the human-like reasoning of fuzzy logic systems with the learning and connectionist of the ANN [23]. The NFS provides powerful and flexible universal approximations with the capability to recognize interpretable IF-THEN rules [24]. The NFS is simply a fuzzy logic system that is trained by a learning algorithm derived from the ANN theory. One of the most important advantages of the ANN is the capability to learn from examples; however, it is hard to prove that the ANN is working as expected. In addition, it is like a “black box” to the user, in which the method for obtaining the output is not revealed [25]. On the other hand, the fuzzy logic system is easy to build and understand by using linguistic expressions to resolve imprecise information [25,26]. However, it is not easy to guarantee that a fuzzy logic system with several complex rules will provide an appropriate degree of meaningfulness. Furthermore, the fuzzy logic system uses static fuzzy rules that lack the adaptability to resolve unpredicted changes in the environment [26].

The integration of the ANN with the fuzzy logic system resolved some of these issues. The resultant NFS combines parallel computation and learning abilities of the ANN with the human reasoning of fuzzy systems and clarity of systems representation. Therefore, the ANN becomes more transparent, and the fuzzy logic system becomes capable of learning [27]. The integration of the ANN with the fuzzy logic system can be done in three ways: cooperative, concurrent, and hybrid. Cooperative NFS is used to describe the integration of the ANN with the fuzzy logic system, in which the ANN is used to tune the fuzzy logic system without changing its functionality [28]. Conversely, concurrent NFS refers to the system where the ANN and the fuzzy logic system work together, in which the inputs entered into the fuzzy logic system are pre-processed, and then the ANN processes the outputs of the concurrent system or in a reverse way [29]. In the hybrid NFS, both fuzzy logic and ANN models are used independently, in which each model is used to perform a certain task in the system to reach a common target [30]. The concept of the hybrid NFS refers to the explanation of the fuzzy logic system with respect to the ANN. Hence, fuzzy sets can be interpreted as weights, and fuzzy rules, input, and output variables can be interpreted as neurons [29].

## 4. Risk-Based Access Control Model

Unauthorized information disclosure is one of the critical challenges in the IoT system that need to be addressed. Current traditional access control models cannot resolve this challenge, since these models are built using static and predefined policies that always give the same result in different situations [15,31]. Therefore, they are not flexible to resolve the varying behaviour of users, especially in a dynamic environment like the IoT. On the other hand, dynamic access control approaches provide an efficient solution for dynamic environments, like IoT, as they utilize not only access policies but also real-time and contextual features to make access decisions.

The risk-based access control model is one of the dynamic models that use the security risk value associated with each access request as a criterion to determine the access decision. It performs a risk analysis to estimate the security risk value for each access request, and then it uses the estimated risk value to decide to either grant or deny access [4,6].

**Figure 1:**
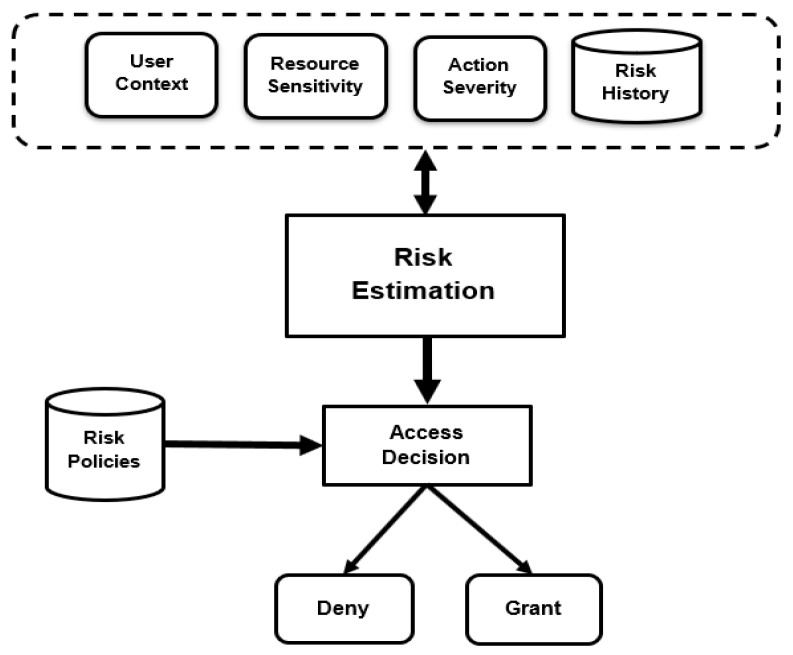
Dynamic risk-based access control mode.

A dynamic risk-based access control model for the IoT was proposed by the authors and discussed in [8,9,32]. The proposed model has four inputs: user/agent context, resource sensitivity, action severity, and risk history, as shown in Figure 1. These inputs/risk factors are used to estimate the security risk value associated with the access request. Then, the estimated risk value is compared against risk policies to specify the access decision. The eventual goal of the proposed risk-based model is to create a system that encourages information sharing to maximize organization benefits while keeping users responsible for their actions.

## 5. Implementation of the Proposed NFS Model

**Figure 2:**
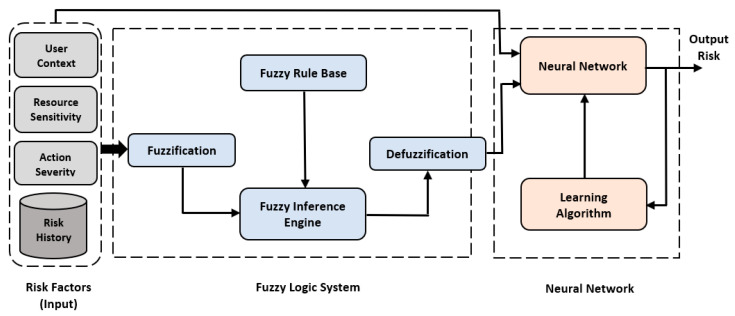
Structure of the Proposed NFS model.

The hybrid NFS was utilized to implement the risk estimation technique of the risk-based access control model. Implementing the hybrid NFS was performed in two separate stages. The fuzzy logic system was first implemented, then the ANN was used to train it. Figure 2 shows the structure of the proposed NFS model. The input involved risk factors: user context, resource sensitivity, action severity, and risk history. Then, the fuzzy logic system was implemented based on the input of IoT security experts [9] to provide a numeric risk value for each input combination. The output of the fuzzy logic system with input was used to build a dataset to train the neural network and select the most appropriate learning algorithm that provided the optimal performance for the risk estimation process. This yielded an accurate output risk value that was used to decide whether to grant or deny access for each access request promptly.

The proposed hybrid NFS model was built by implementing the fuzzy logic system and neural network in two separate phases. In the first phase, the fuzzy logic system was implemented to utilize the effectiveness of converting linguistic expressions into quantitative values, which could be used to produce numeric risk values to make access decisions. The implementation of the fuzzy logic system was discussed in detail in [9]; therefore, we will only summarize it in this paper briefly to show how the entire NFS model was implemented.

To implement a Mamdani Fuzzy Inference System (FIS), there are five main stages, which include fuzzification, MFs, fuzzy rules, rule aggregation, and defuzzification. To determine the required parameters to implement the fuzzy logic system of the risk estimation technique, 20 IoT security experts from inside and outside the UK were interviewed, as discussed in [9].

The first stage was used to convert the input and output variables of the system into linguistic expressions (fuzzy sets), which is called fuzzification. Three fuzzy sets were used to represent each risk factor and five fuzzy sets to represent the output risk. The user context, action severity, and risk history were represented by “Low”, “Moderate”, and “High” fuzzy sets. The resource sensitivity was represented by “Not Sensitive”, “Sensitive”, and “Highly Sensitive” fuzzy sets. Conversely, the output risk was represented by using five fuzzy sets: “Negligible”, “Low”, “Moderate”, “High”, and “Unacceptable High” [9].

The second stage to implement a Mamdani FIS was to specify the MF that represented the relationship between the input risk factors and output risk. A Triangular MF was selected as the appropriate MF to provide a proper representation of the expert knowledge and facilitate the calculation process. The third stage was fuzzy rules, which are the knowledge base that is used by the fuzzy model to generate the output. Fuzzy rules are used to define the relationship between the output risk and input risk factors. It builds input combinations with the corresponding output in the form of IF-THEN statements. As there is no available dataset, there is no way to ensure correct and precise fuzzy rules. Hence, IoT security experts were used to build fuzzy rules based on their knowledge and experience [9]. Since the risk-based model has four inputs/risk factors, each input has three fuzzy sets, and the total number of fuzzy rules was 81 rules.

**Figure 3:**
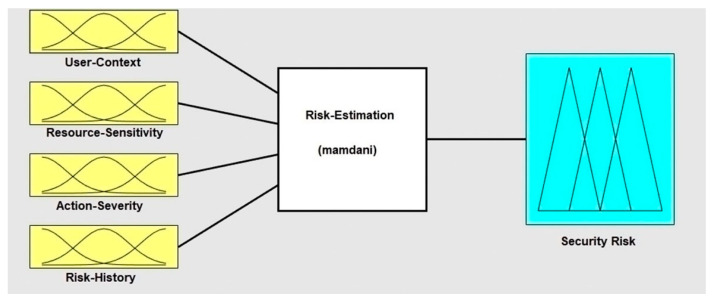
Implementation of Mamdani FIS using the MATLAB fuzzy logic toolbox.

The fourth stage was rule aggregation, which combined the outputs of all fuzzy rules. The max (maximum) aggregation operator was used to combine output rules into one fuzzy set. The fifth and final stage was defuzzification. The output risk of the fuzzy model should be back to be a crisp value. The centroid method was selected to defuzzifize the output. The MATLAB fuzzy logic toolbox was utilized to implement the fuzzy logic system of the risk estimation technique, as shown in Figure 3.

After the fuzzy logic system was implemented, a dataset consisting of 160,000 records was created. Each record contained values of the four risk factors (user context, resource sensitivity, action severity, and risk history) and the output risk value.

**Figure 4:**
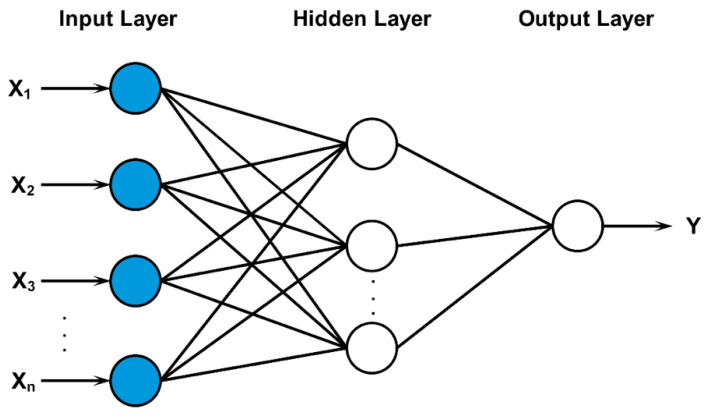
Architecture of an MLP neural network.

The second phase to implement the proposed NFS model was the neural network. A Multi-Layer Perceptron (MLP) model was utilized to implement the neural network. A MLP is a feed-forward neural network that is used to explore complex and nonlinear models. It is based on a supervised learning technique that needs the desired output for each input to be known to calculate the error [33]. The MLP model consists of three layers: the input layer, hidden layer, and output layer, as shown in Figure 4.

The input layer represents the risk factors: user context, resource sensitivity, action severity, and risk history. The output layer represents the output risk value resulting from the risk estimation process. Conversely, the middle layer is the hidden layer that is responsible for carrying out computations and updating weights between different connections. One of the challenges associated with implementing the NFS model was determining the appropriate number of hidden layers and the appropriate number of neurons for each hidden layer. The number of hidden layers needed depends on the complexity of the relationship between the input and the target parameters. It represents a major impact on the learning process. However, a Feed-Forward Back Propagation (FFBP) network encompassing more than one hidden layer is very rare [33]. Hornik, Stinchcombe, and White [34] have proved that an FFBP network with one hidden layer is enough for most problems in various applications. Therefore, one hidden layer was used.

In addition, determining the optimal number of neurons in the hidden layer plays a significant role in the implementation of the NFS model. If an insufficient number of neurons are used, the model will be unable to model complicated data, and the resulting fit will be poor. Conversely, using a large number of neurons in the hidden layer affects its performance on new data, and its ability to provide a generalized model will be compromised [35]. Indeed, increasing the number of neurons ensures correct training, but it also affects performance. Therefore, a compromise needs to be reached between too many and too few neurons in the hidden layer.

In the next section, training the NFS model using various learning algorithms will be discussed to determine the appropriate learning algorithm as well as the appropriate number of neurons in the hidden layer that provides the best performance.

## 6. Experimental Results

Several experiments were carried out to train the proposed NFS model of the risk estimation technique to increase the accuracy of the output risk. Furthermore, several experiments were performed to determine the number of neurons in the hidden layer with various learning algorithms. All training and experiments were coded and executed using the MATLAB software. All experiments and measurements were coded using MATLAB on Intel(R) Core (TM) i7-2600, 3.40 GHz CPU, with 16 GB RAM, running Windows 10.

### 6.1. Data Collection

Implementing the NFS model of the proposed risk estimation technique requires a dataset or examples for training. After implementing the fuzzy logic system, a dataset consisting of 160,000 records was created. To avoid possible bias in the sample data to the NFS, the dataset was randomized and divided into three sets using the cross-validation method.

- Training set: This set contained 96,000 records (60% of the dataset) to train the NFS model.
- Testing set: This set contained 32,000 records (20% of the dataset) to test the NFS model.
- Validation set: This set contained 32,000 records (20% of the dataset) to validate the NFS model.

### 6.2. Performance Evaluation

Commonly used performance evaluation metrics in forecasting problems were utilized to compare and evaluate the accuracy of the NFS model [36]. The NFS model was trained, and the performance was observed using the MSE (Mean Square Error), RMSE (Root Mean Squared Error), and R (Correlation Coefficient). The number of neurons in the hidden layer with the lowest MSE and RMSE and the highest R was selected to implement the NFS model of the risk estimation technique.

(1) $$\mathrm{RMSE}=\sqrt{\frac{1}{n}\sum_{i=1}^{n}(O_{i}-P_{i}{)}^{2}}$$

(2) $$\mathrm{MSE}=\frac{1}{n}\sum_{i=1}^{n}(O_{i}-P_{i}{)}^{2}$$

(3) $$R=\frac{n\sum_{i=1}^{n}O_{i}P_{i}-\sum_{i=1}^{n}O_{i}\sum_{i=1}^{n}P_{i}}{\sqrt{(n\sum_{i=1}^{n}O_{i}^{2}-(\sum_{i=1}^{n}O_{i}{)}^{2})(n\sum_{i=1}^{n}P_{i}^{2}-(\sum_{i=1}^{n}P_{i}{)}^{2})}}$$

where *n* is the total number of data, *O_i_* is the observed value, and *P_i_* is the predicted value.

### 6.3. Training the NFS Model

To reach network generalization and a good fit with all the data points, the proposed NFS model of the risk estimation technique was trained using three learning algorithms, Levenberg–Marquardt (trainlm), Conjugate Gradient with Fletcher–Reeves Resrarts (traincgf), and Scaled Conjugate Gradient (trainscg), to determine the optimal learning algorithm that guarantees network generalization with the minimum error (lowest RMSE and MSE) and the maximum fit (highest R).

#### 6.3.1. Training the NFS with LM

The Levenberg–Marquardt (LM) algorithm is an iterative method that locates a local minimum of a multivariate function. It is expressed as the sum of squares of several non-linear and real-valued functions. The LM algorithm is widely adopted in various domains to deal with data-fitting applications. It has become a standard method for nonlinear least-squares problems. The LM algorithm can be considered as a combination of the steepest descent and the Gauss–Newton method and is one of the fastest learning algorithms [37].

The LM algorithm with one hidden layer was utilized to train the proposed NFS model of the risk estimation technique. Several experiments were carried out to determine the number of neurons that produce the lowest error and the best fit with the learning process. The NFS model was trained using the LM learning algorithm with increasing the number of neurons in the hidden layer from 100 to 1000, and MSE, RMSE, and R values were recorded, as shown in Table 2.

The results demonstrated that increasing the number of neurons in the hidden layer led to decreasing both MSE and RMSE values for training, testing, and validation data and increasing the value of R. The results demonstrated that the NFS model had the lowest MSE and RMSE error values for training, testing, and validation at 1000 neurons. Furthermore, the NFS model had the highest value of R, 0.9985, at 1000 neurons, which is an adequate correlation that indicates the NFS model was well trained and fitted with the learning process, as the value of R is very close to 1.

**Figure 5:**
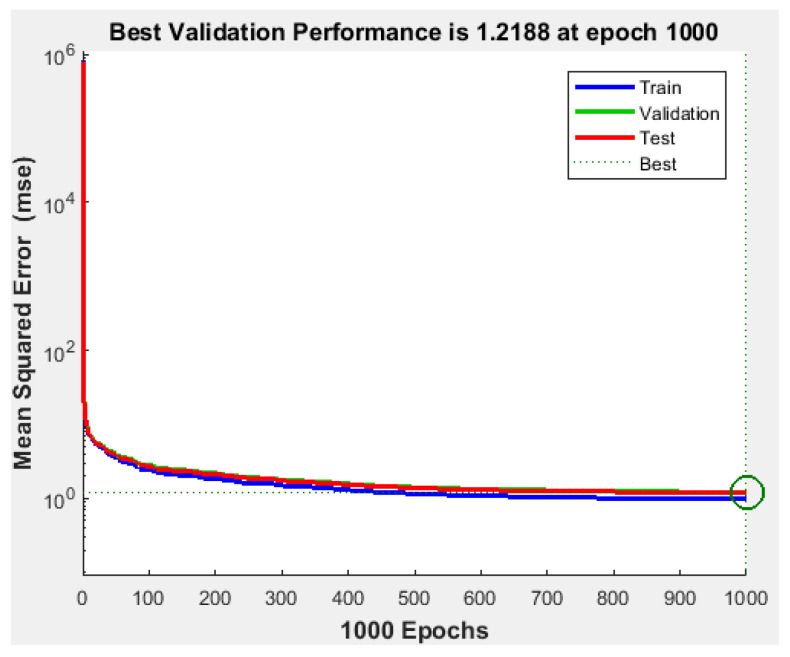
Performance of the training, validation, and testing data with the LM algorithm at 1000 neurons in the hidden layer.

**Figure 6:**
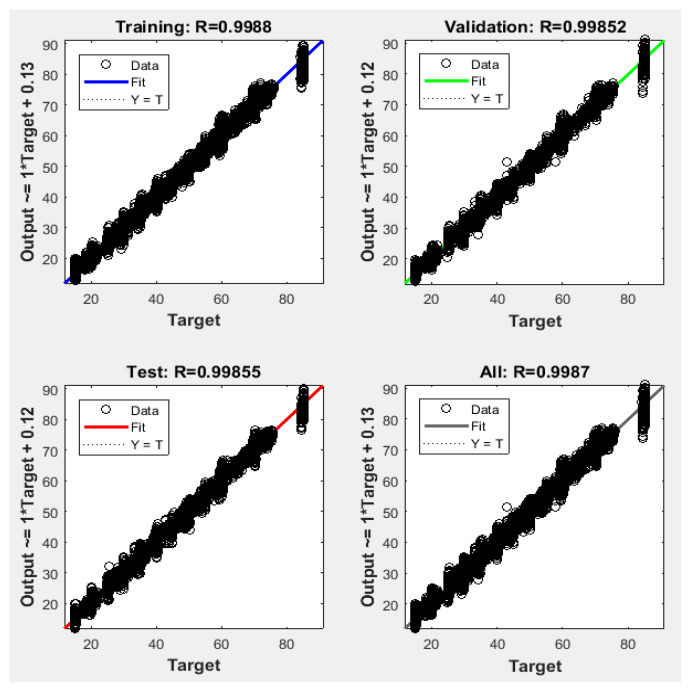
Regression plots of the training, testing, and validation data with the LM algorithm at 1000 neurons in the hidden layer.

Since the best results in terms of MSE, RMSE, and R were obtained using 1000 neurons in the hidden layer, the proposed NFS model was implemented and trained using the LM learning algorithm with 1000 neurons in the hidden layer. The performance graph of the MSE values of training, testing, and validation data is shown in Figure 5. The results demonstrated that the NFS model was a good fit with the learning process, with a value of R of 0.999, which is very close to 1. In addition, no overfitting occurred, as training, validation, and testing data had the same behaviour. In addition, Figure 6 shows regression plots of the proposed NFS model with respect to targets for training, validation, and testing data. For a perfect fit, the data should fall along a 45-degree line, where the network outputs are equal to the targets. For the proposed NFS model of the risk estimation technique, the fit was reasonably good for all training, validation, and testing data with a value of R of 0.999, which is very close to the ideal case.

#### 6.3.2. Training the NFS with CGF

A gradient-based learning algorithm is one of the most commonly used error minimization techniques. It is a gradient descent local optimization algorithm that includes the backward error correction of the network weights [38]. The conjugate gradient algorithm is one of the backpropagation techniques used to train multilayer ANN networks in a supervised way. It updates weight and bias values based on the conjugate gradient backpropagation with Fletcher–Reeves updates [39]. Therefore, it is called the Conjugate Gradient with Fletcher–Reeves (CGF) learning method. The conjugate gradient algorithms are usually much faster than the variable learning rate backpropagation. However, they require more storage than simple algorithms, so they are often a good choice for networks with a large number of weights [40].

The CGF learning algorithm with one hidden layer was utilized to train the proposed NFS model. Several experiments were carried out to determine the number of neurons that produced the lowest error and the best fit with the learning process. The NFS model was trained with increasing the number of neurons in the hidden layer from 50 to 1200, and the MSE, RMSE, and R values were recorded, as shown in Table 3.

The results of training the NFS model using the CGF learning algorithm showed unstable behaviour when increasing the number of neurons in the hidden layer. For example, the MSE value of the training dramatically decreased from 25.27 at 50 neurons to reach 21.04 at 100 neurons. This decrease continued to reach 20.59 at 200 neurons. Then, the MSE value of the training data increased dramatically to reach 26.75 at 300 neurons. Then, the MSE reached its lowest value at 400 neurons. Increasing the number of neurons in the hidden layer from 400 to 1200 showed the same unstable behaviour. However, the MSE value at 400 neurons produced the lowest error. This was the same scenario for the RMSE value for training, validation, and testing data, where it produced the lowest RMSE values at 400 neurons. For the value of R, the results showed the same behaviour of the MSE and RMSE values, in which the lower the error, the higher the correlation. The highest value of R (0.976) for the proposed NFS model was realized at 400 neurons in the hidden layer.

**Figure 7:**
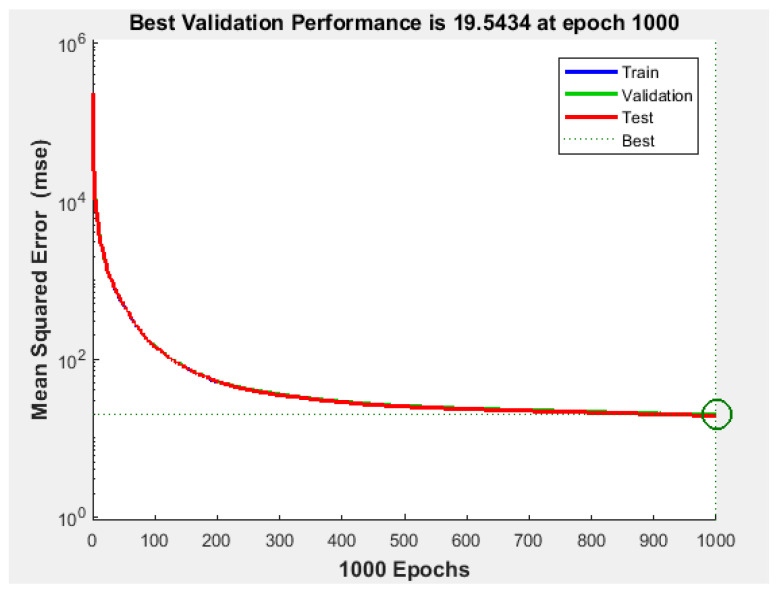
Performance of the training, validation, and testing data with the CGF learning algorithm at 400 neurons in the hidden layer.

**Figure 8:**
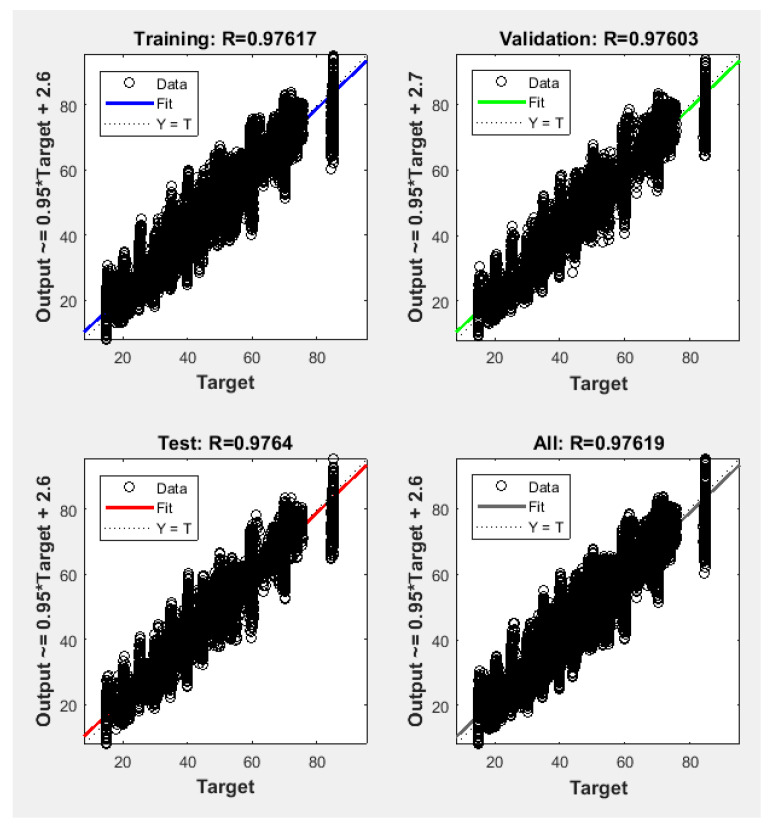
Regression plots of the training, testing, and validation data with the CGF algorithm at 400 neurons in the hidden layer.

Since the best results in terms of MSE, RMSE, and R were obtained using 400 neurons, the proposed NFS model of the risk estimation technique was implemented and trained using the CGF learning algorithm with 400 neurons in the hidden layer. The performance graph of MSE values of training, validation, and testing data is shown in Figure 7. The results demonstrated that the proposed NFS model was a good fit, as the R value was close to 1, as shown in Figure 8. In addition, no overfitting occurred, as the training, validation, and testing data had the same behaviour.

#### 6.3.3. Training the NFS with SCG

Conjugate gradient methods need a line search at each iteration, which is computationally expensive, as it requires that the network response to all training inputs be estimated multiple times for each search. The Scaled Conjugate Gradient (SCG) learning algorithm was developed by Moller in 1993 [41]. It was primarily built to overcome the time-consuming line search associated with conjugate gradient learning methods. The SCG algorithm utilizes the second-order information from the ANN to reach faster convergence. It is also fully automated, so there are no user-dependent parameters, and it avoids a time-consuming line-search in each iteration to determine the appropriate step size [41].

The SCG learning algorithm with one hidden layer was utilized to train the proposed NFS model of the risk estimation technique. The NFS model was trained with increasing the number of neurons in the hidden layer from 50 to 1200, and the MSE, RMSE, and R values were recorded, as shown in Table 4. The results demonstrated unstable behaviour when increasing the number of neurons in the hidden layer. The MSE value of the training data increased from 19.99 at 50 neurons to 23.77 at 100 neurons. Then, the unstable behaviour continued until the MSE reached its lowest value at 1000 neurons. This was the same scenario for the RMSE for training, validation, and testing data, where it produced the lowest values at 1000 neurons. For the value of R, the results demonstrated the same behaviour of MSE and RMSE values, in which the lower the error, the higher the correlation. The highest value of R (0.9975) for the proposed NFS model was realized at 1000 neurons in the hidden layer.

**Figure 9:**
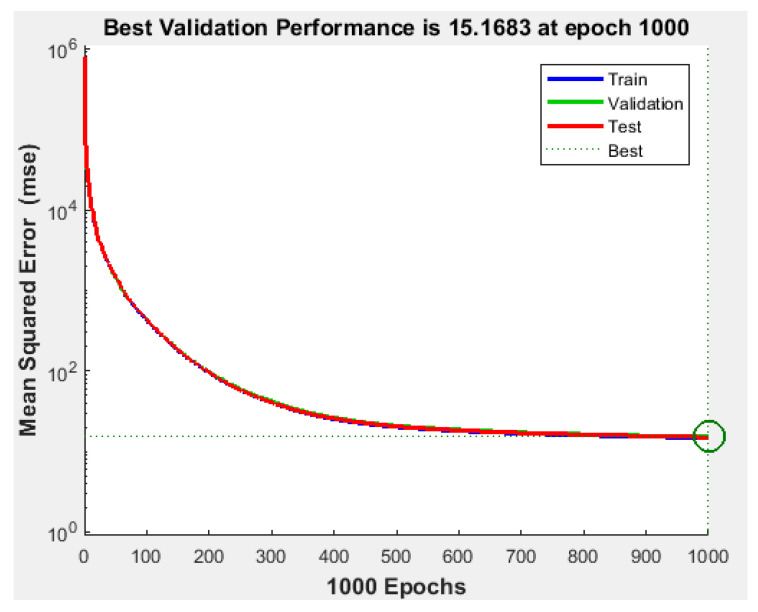
Performance of the training, validation, and testing data with the SCG learning algorithm at 1000 neurons in the hidden layer.

**Figure 10:**
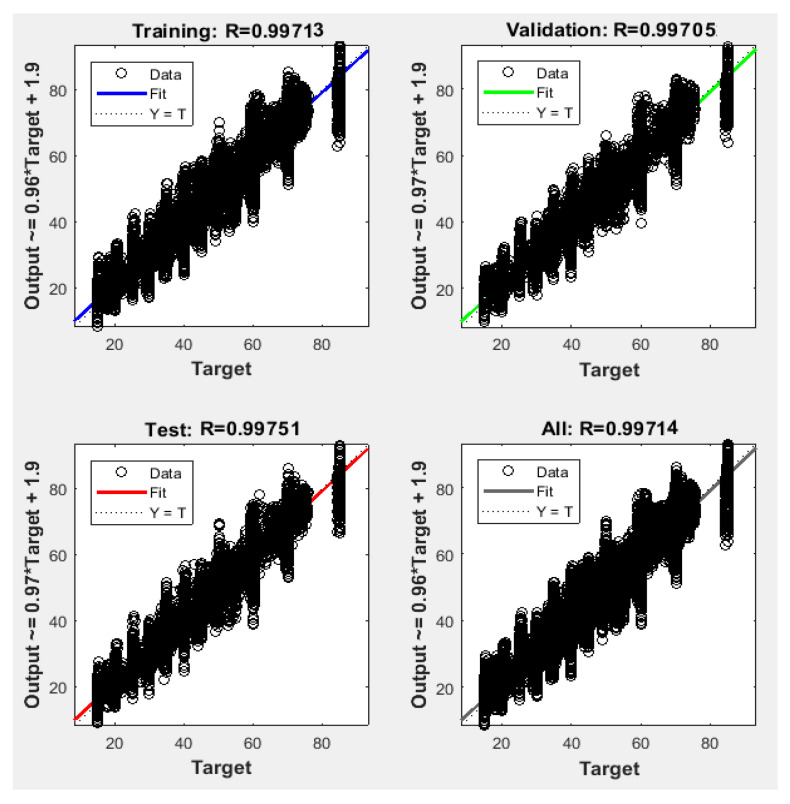
Regression plots of the training, validation, and testing data with the SCG learning algorithm at 1000 neurons in the hidden layer.

Since the best results in terms of the MSE, RMSE, and R were obtained using 1000 neurons, the proposed NFS model of the risk estimation technique was implemented and trained using the SCG learning algorithm with 1000 neurons in the hidden layer. The performance graph of the MSE values for training, validation, and testing data can be shown in Figure 9. The results showed that the NFS model was a good fit, as the value of R (0.9975) was very close to 1, as shown in Figure 10. In addition, no overfitting occurred, as the training, validation, and testing data had the same behaviour.

#### 6.3.4. Comparison of Training Algorithms

A comparison between three learning algorithms that were utilized to train the proposed NFS model is shown in Table 5. The results demonstrated that there was no optimal number of neurons for the hidden layer that could be used to produce the lowest error and the highest correlation with different learning algorithms.

As shown in Table 5, although the MSE and RMSE errors of both the CGF and SCG algorithms were quite high, the correlation of both algorithms was good. This is due to the fact that the CGF and SCG algorithms are mainly designed to avoid the time-consuming line search which produces a good fit in a short period of time [42]. This can be shown by having a good fit (0.976 and 0.997, respectively). In terms of the MSE and RMSE values, the LM learning algorithm produced the lowest error for training, validation, and testing data among other learning algorithms. In addition, the LM algorithm produced the highest correlation, with 0.999 for the value of R, which is close to the ideal case. This indicates that the NFS model was well trained and fitted with the learning process. This is because the LM optimization technique is more powerful than the conventional gradient descent techniques [43]. Therefore, the LM learning algorithm was selected as the optimal learning algorithm to be utilized to implement the proposed NFS model of the risk estimation technique.

### 6.4. NFS and the Fuzzy System

Utilizing the risk-based access control model to decide whether granting or denying access needs a risk estimation technique that produces a quantitative and numeric risk value for each access request. However, there was no available dataset to describe the risk probabilities and impacts for a set of specified incidents. In the absence of a dataset, the fuzzy logic system using the Mamdani FIS was utilized with the input of IoT security experts to implement the risk estimation process [9]. One of the challenges that faced adopting the fuzzy logic system in the risk estimation technique in real-world IoT applications is that it requires a long processing time, and its scalability seems to be questionable. To overcome these issues, the risk estimation technique was implemented using the hybrid NFS model. This is because the hybrid NFS model makes the best use of the advantages of fuzzy systems and ANNs. Hybrid systems have the property of constantly being treated as systems of fuzzy rules, with neural networks used to tune MFs in the preconditions and conclusions of rules based on the set of learning [44]. Furthermore, the parallel computation and learning abilities of the NFS model add more improvements to the risk estimation technique.

After training the NFS model using three learning algorithms (LM, CGF, and SCG), the results demonstrated that implementing the proposed NFS model with the LM learning algorithm to implement the risk estimation technique provided less processing time, as it used only one-sixth of the time used by the Mamdani FIS, as depicted in Table 6. Both methods followed a linear relationship, in which increasing the number of access requests led to increasing the processing time. In addition, the results demonstrated that the time per access request for the NFS model using the LM algorithm produced a very short time compared to the time per access request produced by the Mamdani FIS. As shown in Table 6, the processing time required to process 1000 access requests using the Mamdani FIS was 57.38 s, while it only took 10.87 s when processed using the proposed NFS model with the LM learning algorithm. The trained NFS model with the LM learning algorithm proved that it provides a more efficient processing time, which can provide timeliness risk estimation techniques for various IoT applications. Besides this, the learning capability makes the risk estimation technique able to adapt to changes and unpredicted situations in the IoT environment, which will result in more accurate and realistic risk values.

## 7. Evaluation of Results: Healthcare

Healthcare is one of the main applications of the IoT system [45,46]. It is used to provide remote monitoring and constant tracking of health conditions, which provide a more effective healthcare system for various patients. Due to the vast volume of sensitive data collected by various healthcare devices about patients, that if accessed by unauthorized individuals can lead to severe and deadly consequences, providing a secure, dynamic, and flexible access control model that uses not only access policies but also real-time and contextual features to provide access decisions is required [47,48]. In addition, protecting patients’ data is not the only concern in healthcare systems but also regarding providing access in unexpected situations. In crises or emergencies, the availability of information takes precedence over privacy and security concerns. Therefore, providing a dynamic access control model for healthcare is a significant aspect to ensure data security and adapt to unexpected situations [49]. Hence, the healthcare scenario was utilized to check the applicability of the proposed risk-based access control model using the NFS model for the risk estimation.

This section discusses applying the proposed risk-based access control model and the risk estimation technique using the NFS model. Different access control scenarios will be presented to evaluate the applicability of the proposed model to real-world scenarios.

### 7.1. Scenario Description

A closed world scenario involving a healthcare provider, such as Mount Cedar (MC) children’s hospital [50], was utilized to show various access control scenarios. Typically, patients’ information in hospitals is stored as datasets. Each dataset is characterized by a unique object identifier. Datasets can be organized in classes that can be collectively referred to with a given name and associated with an object profile (metadata) that provides additional information about the dataset.

Consider the MC hospital has now received a four-year-old child called Harry, who was brought into the MC’s first aid clinic by his mother, Eva, late Wednesday evening. The admitting staff observed that Harry suffered from several bruises all over his body, a fractured rib, and a distorted shoulder [50]. Let us walk through the events that would occur in this situation. Initially, Harry’s doctor in the first aid clinic, Dr Chris, made an access request to the system to view or read Harry’s history file in the Electronic Patient Record (EPR). He also assigned Harry to a care team involving a set of nurses and ordered a series of examinations. The leader nurse of the care team made an access request to the system to read Harry’s file in the patient’s EPR.

When the examination results returned, Dr Chris wrote the diagnosis and the required medication for Harry and called social workers and policemen to investigate the incident, as he suspected a child abuse has occurred. Therefore, one of the social workers who is responsible for helping the children in case of abuse and a police officer requested to access Harry’s medical information for investigation purposes.

### 7.2. Scenario Actors

The MC is a children’s hospital. Actors involved in this scenario include:

- The child who needs treatment;
- Doctors who are responsible for providing care to the child;
- Nurses who are responsible for helping the doctors;
- Social workers who are responsible for helping the children in case of trauma or abuse;
- Policemen who are responsible for investigating and establishing possible criminal charges and responsibilities in cases of trauma or abuse.

### 7.3. Scenario Assumption

There is no available dataset that describes risk likelihood and its impact nor has similar work done before to use the NFS model in the risk estimation in risk-based access control models. Some parameters will be assumed based on the literature review and logical flow of the scenarios. Applying the proposed risk-based access control model on the healthcare access control scenario requires defining values of the four risk factors (action severity, resource sensitivity, user context, and risk history) for each access request. For the action severity, three actions were assumed, involving read/view, write, and delete. The delete operation is not permitted for all actors involved in this scenario, as the hospital keeps track of all the medical histories of patients, so there is no need to delete any data. As discussed earlier, there are various actors involved in this scenario, in which each actor has a different role in the hospital. The proposed risk-based model should validate its applicability in this scenario by allowing or denying tasks for each role. Generally, only doctors can perform both read and write operations on the EPR, while other actors, including nurses, social workers, and policemen, can only read/view the EPR. For the resource sensitivity, two sensitivity levels were assumed: sensitive and not sensitive. However, all data/resources involved in this scenario were assumed to be sensitive.

To define the value of the action severity, Sharma et al.’s [51] formula was utilized. This formula was used to estimate the risk score of action severity in terms of various actions, risk probability, and cost regarding data availability, integrity, and confidentiality. The formula is represented as:

Risk = (C × P) + (I × P) + (A × P)(4)

where C, I, and A represents confidentiality, integrity, and availability, respectively, and P represents the probability. In addition, Sharma et al. [51] have suggested some actions and corresponding values of the CIA, as shown in Table 7. Therefore, values of action severity of the proposed risk-based model will be estimated using this table.

As shown in Table 7, if a user needed to perform a “view” operation on sensitive data, the probability of this incident was 0.2. Since only confidentiality would be affected (second row), the risk value of the action severity would be 0.2. Since healthcare data contain sensitive information, that if used maliciously can lead to deadly consequences, all data or resources involved in this scenario were assumed to be sensitive and with the probability of 0.2, and the value of the resource sensitivity was 0.4. For the contextual and real-time attributes (user context) that were collected at the time of the access request, the time and location features were utilized. The time refers to the time of duty for the hospital staff, whether a doctor or nurse, in which if the doctor requested access to data during his/her time of duty (time allocation), the risk associated with the time context feature would be low, otherwise it would be high. Furthermore, the location was utilized to determine the risk associated with contextual attributes, in which if the actor requested access to data from inside the hospital, the risk would be low, otherwise, the risk would be high. The value of user context was assumed, as shown in Table 8. In addition, since actors involved in this scenario, involving doctors, nurses, social workers, and policemen, are officially employed in the hospital, they are trusted users, and hence their risk history was assumed to be low. Therefore, the value of the risk history would be 0.2.

### 7.4. Scenario Results

The output risk value was used to assess the security risk value associated with the access request and used to make the access decision. The risk values of this scenario were categorized into three groups, as shown in Table 9. The security administrator could utilize these bands to grant or deny access to system resources. For example, if the output risk is low or moderate, access can be granted, while if the output risk is high, the access should be denied. These bands can be flexibly changed by the security administrator in response to unpredicted situations. Furthermore, the security administrator or owner has the full flexibility to specify different values for the risk category and specify their output risk band to grant or deny access.

All access control scenarios of the MC children’s hospital were implemented, and the output risk value was evaluated using the proposed NFS model with the LM algorithm, as shown in Table 10. Applying the proposed risk-based access control model and the risk estimation using the NFS model demonstrated that it can provide several advantages to the healthcare domain. Using contextual and real-time features involving time and location demonstrated it can provide dynamic and flexible access decisions that can adapt to unpredicted situations. Allowing the doctors to access the patient’s EPR even after finishing their duty time allows them to help the patient until an available on duty doctor is allocated. In addition, one of the important aspects of applying the proposed risk-based model in the healthcare domain is denying access, whether to reading or writing operations, for all actors involved in this scenario when they are not on duty and outside the location of the hospital. This adds more security to the healthcare system compared to the existing systems, in which if one actor lost his/her credentials (for example password) through social engineering or any other type of attack, this can lead to information disclosure. Using the proposed risk-based model with contextual and real-time features, no-one can access data only if they are inside the hospital and within their duty time.

Applying the proposed risk-based model on access control scenarios of the MC children’s hospital demonstrated it can provide an effective access control model that can use contextual and real-time features to provide access decisions. It solves issues associated with static policies that always give the same result in different situations. For example, it allows access if the actor is located in the hospital location. It also solves issues associated with misuse and credential loss by allowing access by actors in person.

## 8. Conclusions

A risk-based access control model is one of the dynamic models that utilizes real-time and contextual features to make access decisions. This model performs a risk analysis on each access request to permit or deny access dynamically based on the estimated risk value. The risk estimation process is one of the essential stages to implement a risk-based access control model for the IoT. Although the fuzzy logic system provides accurate and realistic risk values for access control operations, it has some limitations. For example, the scalability of the fuzzy logic system seems to be doubtful, since it requires a non-trivial time to estimate security risks of access control operations. Furthermore, the fuzzy logic cannot learn or adjust itself to a new environment. Therefore, this paper proposed a novel NFS model to build the risk estimation technique that evaluates security risks associated with access requests. The proposed NFS model was trained using three learning algorithms, LM, CGF, and SCG, to determine the optimal learning algorithm with the minimum error (lowest RMSE and MSE) and the maximum fit (highest R). The results demonstrated that the LM algorithm was the optimal method to implement the NFS model of the risk estimation process. The trained NFS model with the LM learning algorithm proved that it provides a more efficient processing time, which can provide a timeliness risk estimation technique for various IoT applications. Besides this, adding the learning capability will make the risk estimation technique able to adapt to changes in the IoT environment, which results in more accurate and realistic risk values. The proposed NFS model was also evaluated against access control scenarios of a healthcare system (children’s hospital), and the results demonstrated that the proposed NFS model provides dynamic, flexible, and accurate access decisions using the contextual and real-time features involving the time and location associated with each access request. For future work, deep learning techniques will be investigated for the risk estimation process. With the availability of a large dataset in this research, deep learning algorithms are expected to provide better results in terms of accuracy and performance. In addition, investigating utilizing risk with user trust to provide access decisions should be performed.

## Figures and Tables

**Table 1:** Summary of related work.

Citation	Summary of Contribution
McGraw [10]	Proposed an RAdAC model that provides access based on security risks and operational needs. However, the model does not provide details about how to estimate security risks and operational needs quantitatively.
Khambhammettu et al. [11]	Proposed a framework to assess security risks based on object sensitivity and subject trustworthiness. However, the model lacks contextual features and is unable to learn or adapt to unpredicted situations.
Chen et al. [12]	Proposed a dynamic risk-based model that integrates the attribute-based access control model with the risk-trust assessment method. However, the model lacks contextual features and is unable to learn or adapt to unpredicted situations.
Choi et al. [13]	Proposed a context-sensitive risk-based model that uses action severity and treatment to provide access decisions. However, the model does not include how to estimate the risk quantitatively.
Chen et al. [5]	Proposed a fuzzy multi-level security model to estimate security risks using the difference between the object and subject security levels. However, the model does not explain how to estimate risk quantitatively. Furthermore, time overhead and scalability issues have not been addressed.
Ni et al. [14]	Proposed a fuzzy logic system to assess security risks based on the subject and object security levels. However, the model does not explain how fuzzy rules were created or how scalability and time-overhead issues can be addressed.
Li et al. [15]	Proposed a fuzzy modelling technique to assess security risks based on action severity, risk history, and data sensitivity. However, the model does not use contextual features.
Atlam et al. [9]	Proposed a fuzzy logic system to assess security risks based on action severity, risk history, resource sensitivity, and user context. However, the model lacks the ability to learn or adapt to unpredicted situations.
Diep et al. [16]	Proposed a dynamic risk-based model by collecting useful information from the environment to make access decisions based on outcomes of actions. However, the model does not cover how values of outcomes of actions can be evaluated.
Kristjanpoller & Michell [19]	Proposed the NFS model to assess security risks of a stock market in the Latin American region and improve the forecast accuracy.
Beinarovica et al. [20]	Proposed the NFS model in transportation control to improve safety by evaluating the risk of accidents using intelligent infrastructure devices.
Shahzadi et al. [21]	Proposed the NFS model to reduce security risks in cloud computing by identifying various risks associated with it.
Kaur et al. [22]	Proposed the NFS model to improve authentication in mobile devices by building an implicit authentication system based on behavioural data collected from different android users.

**Table 2:** MSE, RMSE, and R values when training the NFS model using the LM algorithm.

Number of Neurons	Training MSE	Validation MSE	Testing MSE	Training RMSE	Validation RMSE	Testing RMSE	R
100	6.299	6.492	6.472	2.509	2.548	2.544	0.9921
200	4.1903	4.3934	4.4112	2.0470	2.0960	2.1003	0.9946
300	3.0523	3.3245	3.2608	1.7471	1.8233	1.8058	0.9960
400	2.2228	2.4041	2.4442	1.4909	1.5505	1.5634	0.9970
500	1.9234	2.1845	2.1624	1.3869	1.4780	1.4823	0.9973
600	1.6237	1.7832	1.7576	1.2742	1.3354	1.4237	0.9976
700	1.4456	1.6810	1.6344	1.2023	1.2965	1.2785	0.9980
800	1.2196	1.4294	1.4971	1.1044	1.1956	1.2236	0.9983
900	1.1031	1.3154	1.2491	1.0376	1.1477	1.1546	0.9984
1000	0.9777	1.2188	1.1902	0.9888	1.1040	1.0910	0.9985

**Table 3:** MSE, RMSE, and R values when training the NFS model using the CGF algorithm.

Number of Neurons	Training MSE	Validation MSE	Testing MSE	Training RMSE	Validation RMSE	Testing RMSE	R
50	25.2682	25.3047	25.7503	5.0267	5.0304	5.0745	0.9677
100	21.0426	21.1199	20.8287	4.5872	4.5956	4.5638	0.9744
200	20.5918	20.6355	20.3387	4.5378	4.5426	4.5098	0.9747
300	26.7536	26.7756	27.2466	5.1724	5.1745	5.2198	0.9663
400	19.2530	19.5434	19.1358	4.3878	4.4208	4.3745	0.9764
500	19.6103	19.6805	19.8924	4.4284	4.4363	4.4601	0.9756
600	23.2187	23.3848	23.6404	4.8186	4.8358	4.8621	0.9706
700	22.6813	22.5313	23.1297	4.7625	4.7467	4.8093	0.9715
800	20.6374	21.3769	20.8723	4.5428	4.6235	4.5686	0.9741
900	20.8324	21.1024	20.9875	4.5643	4.5937	4.6275	0.9740
1000	22.6382	23.5947	23.2892	4.7580	4.8574	4.8259	0.9710
1100	24.9580	25.6822	26.0088	4.9958	5.0678	5.0999	0.9679
1200	21.0426	21.1199	20.8287	4.5872	4.5956	4.5638	0.9744

**Table 4:** The MSE, RMSE, and R values when training the NFS model using the SCG algorithm.

Number of Neurons	Training MSE	Validation MSE	Testing MSE	Training RMSE	Validation RMSE	Testing RMSE	R
50	19.9948	20.3018	20.5614	4.4716	4.5058	4.5345	0.9745
100	23.7679	23.9127	24.0920	4.8752	4.8901	4.9084	0.9704
200	17.8191	18.0397	18.1198	4.2213	4.2473	4.2567	0.9780
300	20.8936	20.8575	21.2434	4.5710	4.5670	4.6091	0.9735
400	16.7471	17.2101	16.9645	4.0923	4.1485	4.1188	0.9791
500	18.9167	18.9898	19.4518	4.3493	4.3577	4.4104	0.9759
600	21.1014	21.5886	21.3624	4.5936	4.6464	4.6219	0.9738
700	18.6752	18.6925	19.2815	4.3215	4.3235	4.3911	0.9763
800	18.9898	19.6473	19.2034	4.3577	4.4325	4.3822	0.9762
900	18.1027	18.2766	18.2557	4.2547	4.2751	4.2727	0.9815
1000	14.7502	15.1683	15.0113	3.8406	3.8947	3.8744	0.9975
1100	18.9358	19.3698	19.3034	4.3515	4.4011	4.3936	0.9763
1200	19.8946	20.8215	21.3234	4.4603	4.5631	4.6177	0.9746

**Table 5:** Comparison between the learning algorithms used to train the proposed NFS model.

Item	Training Algorithms		
	LM	CGF	SCG
Number of neurons in the hidden layer	1000	400	1000
Number of epochs	1000	1000	1000
MSE of training	0.978	19.253	14.750
MSE of validation	1.219	19.543	15.168
MSE of testing	1.190	19.136	15.011
RMSE of training	0.989	4.388	3.841
RMSE of validation	1.104	4.421	3.895
RMSE of testing	1.091	4.375	3.874
Correlation Coefficient (R)	0.999	0.976	0.997

**Table 6:** Processing time of the NFS model using the LM learning algorithm and Mamdani FIS.

Number of Access Requests	NFS Using the LM Algorithm		Mamdani FIS [9]	
	Time (s)	Time per Request (s)	Time (s)	Time per Request (s)
1000	10.8750	0.01088	57.385	0.0574
10,000	81.5469	0.00815	572.125	0.0572
20,000	146.5625	0.00733	1140.4	0.05702
30,000	211.4216	0.00705	1713.6	0.05712
40,000	277.6094	0.00694	2286.4	0.05716
50,000	341.7656	0.00684	2860.5	0.05721
60,000	407.1875	0.00679	3436.2	0.05727
70,000	472.1250	0.00674	4012.4	0.05732
80,000	537.2345	0.00672	4588.8	0.05736
90,000	602.2314	0.00669	5166.9	0.05741
100,000	667.1286	0.00667	5746.23	0.05746
150,000	995.4688	0.00664	8625.32	0.0575
200,000	1325.3124	0.00663	11,506.14	0.05753
250,000	1634.8213	0.00654	14,390.1	0.05756

**Table 7:** Risk values associated with action and data sensitivity.

Action	Sensitivity	C	I	A
Create	Sensitive/Not-Sensitive	0	1	1
View	Sensitive	1	0	0
View	Not-Sensitive	0	0	1
Modify	Sensitive/Not-Sensitive	0	1	1
Delete	Sensitive/Not-Sensitive	0	1	1

**Table 8:** The risk value of user context of various actors involved in this scenario.

Actor	On Duty (Time)	Location (In Hospital)	User Context Risk Level	Proposed UC Value
Doctor	Yes	Yes	Low	0.2
	No	Yes	Moderate	0.4
	No	No	High	0.7
Nurse	Yes	Yes	Low	0.2
	No	Yes	Moderate	0.4
	No	No	High	0.7
Social Worker	Yes	Yes	Low	0.2
	No	Yes	Moderate	0.4
	No	No	High	0.7
Policeman	Yes	Yes	Low	0.2
	No	Yes	Moderate	0.4
	No	No	High	0.7

**Table 9:** Proposed output risk bands for the scenarios.

Risk Band	Risk Category
0.1–0.3	Low
0.31–0.5	Moderate
0.51–1.0	High

**Table 10:** Access decisions of various scenarios of the MC children hospital.

Actor	On Duty	In Hospital	Action	Risk Factors				Output Risk	Output Risk Category
				UC	RS	AS	RH		
Doctor	Yes	Yes	Read	0.2	0.4	0.2	0.2	0.277	Low
	No	Yes	Read	0.4	0.4	0.2	0.2	0.353	Moderate
	No	No	Read	0.7	0.4	0.2	0.2	0.533	High
	Yes	Yes	Write	0.2	0.4	0.4	0.2	0.338	Moderate
	No	Yes	Write	0.4	0.4	0.4	0.2	0.412	Moderate
	No	No	Write	0.7	0.4	0.4	0.2	0.581	High
Nurse	Yes	Yes	Read	0.2	0.4	0.2	0.2	0.277	Low
	No	Yes	Read	0.4	0.4	0.2	0.2	0.353	Moderate
	No	No	Read	0.7	0.4	0.2	0.2	0.533	High
Social Wo.	Yes	Yes	Read	0.2	0.4	0.2	0.2	0.277	Low
	No	Yes	Read	0.4	0.4	0.2	0.2	0.353	Moderate
	No	No	Read	0.7	0.4	0.2	0.2	0.533	High
Policeman	Yes	Yes	Read	0.2	0.4	0.2	0.2	0.277	Low
	No	Yes	Read	0.4	0.4	0.2	0.2	0.353	Moderate
	No	No	Read	0.7	0.4	0.2	0.2	0.533	High
